# Prevalence of genital high-risk human papillomavirus infections and associated factors among women living with human immunodeficiency virus in Uganda

**DOI:** 10.1186/s12885-024-11928-0

**Published:** 2024-02-21

**Authors:** Harriet Nakigozi, Rawlance Ndejjo, William Bazeyo, Annet Nabaggala, Caroline Achola, Moses Iga, Simeon Kalyesubula, Ben Kanamwangi, Gerald Mutungi, Charles Batte, David Mukunya, Lawrence Sserwanga, Godfrey Gemageine, Charles Akiya Oyoo, Susan Nabadda

**Affiliations:** 1 National Health Laboratory and Diagnostic Services, Ministry of Health, Kampala, Uganda; 2 Department of Disease Control and Environmental Health, School of Public Health, College of Health Sciences, Makerere University, Kampala, Uganda; 3 Department of Non-communicable Diseases, Ministry of Health, Kampala, Uganda; 4 Department of Medicine, Lung Institute, School of Medicine, College of Health Sciences, Makerere University, Kampala, Uganda; 5 Faculty of Health Sciences, Department of Community and Public Health, Busitema University, Mbale, Uganda

**Keywords:** Human immunodeficiency virus (HIV), Human papillomavirus (HPV), Cervical Cancer, Cervical Cancer screening, HPV Aptima, Hologic, Panther, Preservecyt solution

## Abstract

**Background:**

Women living with HIV are at risk for cervical dysplasia and cancer worldwide. In 2015, the World Health Organization (WHO) recommended that testing for high-risk HPV (hrHPV) infection be incorporated into cervical cancer screening programs using molecular nucleic acid tests (NATs) but this has not previously been done in Uganda. The country’s coverage for Human Papilloma Virus (HPV) screening remains low at less than 10% for women aged 25–49 years. This study determined the genital prevalence of hrHPV infection and the associated factors among women living with HIV in Uganda.

**Methods:**

A descriptive cross-sectional study was conducted in 15 selected health facilities among participants who were on Antiretroviral therapy (ART). Participants who consented to participate were instructed on how to collect their own high vaginal swabs using a cervical brush for HPV molecular testing (HPV DNA or HPV RNA) and their demographics data was collected using a standard questionnaire. Laboratory diagnosis for HPV molecular testing was done using Gene xpert machines and Hologic Aptima Machine. Modified Poisson regression analysis was conducted to determine the associated factors.

**Results:**

This study involved 5856 HIV positive participants on ART. A total of 2006 out of 5856 (34.3%) participants had high risk HPV infections. HPV infections by genotypes were: HPV16 317(15.8%), HPV 18/45 308 (15.4%) and other high-risk HPV 1381 (68.8%). The independent factors associated with all hrHPV were parity, education level, having more than one partner, and engaging in early sex. Smoking was associated with HPV 16, HPV 18/45 and other hrHPV. Age was associated with all hrHPV, marital status with HPV 16, and occupation with HPV 16.

**Conclusions:**

The prevalence of genital high-risk HPV infections among HIV positive women attending ART clinics in public facilities in Uganda was high. Other hrHPV genotype was the commonest compared to 18/45 and HPV 16. The integration of cervical cancer screening in ART programmes remains paramount to support the early detection of cervical cancer and Non-invasive self-collected urine and vaginal sampling for cervical cancer screening present an opportunity.

## Background

Persistent infection with high-risk types of human papillomavirus (hrHPV) is a cause of cervical cancer [1]. Human Immunodeficiency Virus (HIV) has complex effects on female genital HPV, which include increased risks of infection of 3–5 times [2–4]. Despite being a largely preventable and curable disease, cervical cancer is the fourth leading cause of cancer death among women accounting for 570,000 diagnoses and 311,000 deaths each year worldwide [5].

The prevalence and distribution of genital hrHPV infections is attributed to factors such as poverty, HIV, high parity, promiscuity, smoking and multiple sexual partners [6]. In developing countries, access to screening, diagnosis and treatment is extremely limited resulting in late presentation and additional demands on the already stretched tertiary centres [3, 7].

In 2018, Africa had the 20 highest cervical cancer burdened countries with the East African region having the highest burden with an incidence of 40.1 per 100,000 and a mortality rate of 28.6 per 100,000 [6]. Cervical cancer is a major public health concern in Uganda because of high mortality rates that are due to late diagnosis [8]. More than 80% of Ugandan women with cervical cancer are not diagnosed until the disease has progressed to an advanced stage that is challenging to treat effectively thus leading to death [9]..

Uganda’s HIV prevalence is 6.2% [9] with 1.4 million people living with HIV, higher in women [10, 11]. The combination of a high HIV rate and late cancer detection has resulted in lower treatment success and survival for Ugandan women with cervical cancer.

A number of studies in select districts of Uganda have documented a high prevalence of 73.5% for human papillomavirus (HPV) types 16 and 18. The remarkably high rates of these cancer-linked HPV types in surveyed regions of Uganda further demonstrate the significant cervical cancer burden in the country [3, 7]..

The lack of a comprehensive cervical cancer prevention programme, diagnosis or screening for HPV oncogenic genotypes or high risk genotypes has resulted in a high burden and increasing cases of cervical cancer in Uganda [12]. Therefore, this study determined the genital prevalence of hrHPV infection and the associated factors among women living with HIV in Uganda.

## Methods

### Study design

This cross-sectional study was conducted in 15 public health facilities across four regions of Uganda that provide antiretroviral therapy (ART) services. The facilities included 12 regional referral hospitals (Mubende, Naguru, Masaka, Mbarara, Kabale, Soroti, Mbale, Hoima, Fort Portal, Gulu, Lira) and 3 general hospitals (Lacor, TASO Jinja, Kyenjojo). They were selected based on having a high volume of patients on ART, a functional laboratory, and mother-baby care services. To offer HPV DNA testing, 12 sites with GeneXpert platforms and trained staff were chosen. The remaining 3 sites were selected to provide HPV RNA testing using Hologic Panther or Aptima Assay systems. By conducting the study across multiple regions at referral facilities with strong ART and lab capacity, the aim was to recruit a large sample of HIV positive women representative of different geographies in Uganda.

### Study population

The study population consisted of HIV positive women aged 25–49 years who were attending routine antiretroviral therapy (ART) clinics. Exclusion criteria included women who were actively menstruating, had given birth within the past three months, or had undergone total hysterectomy for benign conditions. By focusing on HIV positive women in this age group receiving ART, the study aimed to examine women who would be most likely to benefit from HPV screening as part of cervical cancer prevention efforts.

### Sample size

The sample size was determined based on the estimated prevalence of HPV among HIV positive patients in the general Ugandan population. Furthermore, we used the Rao (1985) [13] for a field survey to estimate the prevalence rate of specific event or cases or disease the sample size is calculated by the formula: *n* = 4 p q / L^2^.

With an assumed HPV prevalence of 21% [14], a 5% margin of error, 20% allowance for incomplete data or withdrawal, and a 95% confidence level (CI). Therefore, we were able to get a target sample size of 5856 participants. This sample size estimation ensured that the study would have sufficient statistical power to estimate the population prevalence of HPV among HIV positive women in Uganda within 5% points and a 95% CI, after accounting for possible missing data or dropouts. Published statistics on the number of HIV positive ART patients in Uganda were used to inform what sample size would be feasible and adequately precise based on the population prevalence.

### Sampling procedure

This study was conducted in the ART clinic and enrolled all patients with age range of 25–49 years who consented to participate and were active on ART in the 15 public facilities. These included Fortportal, Gulu, Hoima, Kabale, Lira, Masaka, Mbale, Mbarara, Moroto, Mubende and Naguru regional referral hospitals that had GeneXpert machines on site. Then Lacoh, Kyenjojo and TASO Jinja were general hospitals that sent the collected samples to the central public health laboratories (CPHL) that had the HPV RNA Hologic assay.

### Data collection

Quantitative data was collected using a semi-structured questionnaire administered by trained research assistants through in-person interviews. Eligible participants were consecutively enrolled from ART clinics and mother-baby care points after receiving education on cervical cancer screening. The questionnaire gathered information to assess eligibility criteria such as parity and active menstruation.

Participants were then instructed on collecting a self-vaginal sample for HPV testing using a cervical brush. For privacy, they were taken to a designated medical room or area at the facility. Following a 10-minute demonstration, participants collected their own sample using the brush per manufacturer guidelines and pictorial instructions. The brush was immediately placed back in the container and returned to the health worker. The samples were transported to the on-site laboratory by health workers who recorded the expected turnaround time for results. This process allowed quantitative data to be systematically collected from a large sample of women using a uniform technique and standardized instructions to support self-collection of vaginal samples for HPV testing.

### Sample processing

Once received by laboratory staff, the vaginal samples collected on the brushes were immersed in vials containing PreservCyt medium. The vials were vortexed for 3 × 15 s to ensure complete immersion of the sample. Then, 1 ml of the PreservCyt sample was transferred to Xpert® HPV cartridges and tested using the GeneXpert platform following standard operating procedures for the assay. This laboratory process allowed for standardized HPV DNA testing across the study sites equipped with GeneXpert machines.

The use of PreservCyt ensured proper sample preservation and processing, while the GeneXpert HPV assay provided sensitive and specific detection of high-risk HPV DNA [15]. All samples were tested within 24 h, where possible. Laboratory data for HPV molecular testing was obtained from Genexpert machines located at public health facilities and Hologic or panther testing platforms located at National Health Laboratories and Diagnostic Services at the Ministry of Health. These provided results to determine the prevalence of HPV infections or cervical cancer related infections in HIV positive women.

### Diagnosis of hrHPV infections

After the sample preparation, sample were inserted into the HPV DNA genexpert assay for facilities that were using it or into the HPV RNA Hologic assay for facilities that were using it. This detected active HPV infection and these were categorized into three groups that is HPV 16, HPV18/45 and other hrHPV.

### Using GeneXpert machine

This method was used to test 65.3% (3824/5856) samples which were mainly from the regional referral hospitals. Detection of high-risk HPV was carried out using a DNA molecular testing platform referred to as a genexpert. Genexpert machines and reagents are manufactured by Cepheid a USA based company found in Sunnyvale, California. It uses fluorescence to detect the presence of the high risk or (oncogenic) types of HPV such as HPV 16, HPV 18/45 and other high-risk HPV (hrHPV) in a single run [16].

Genexpert HPV assay detects hrHPV infections of the following types: HPV 16, HPV 18/45; and reports 11 other high-risk types in pooled results in less than one hour. The Xpert HPV Assay is a qualitative in vitro test for the detection of the E6/E7 region of the viral DNA genome from 14 high risk HPV types in a single analysis. Genexpert HPV assay specifically identifies types HPV16 and HPV 18/45 in two distinct detection channels, and reports 11 other High-risk types (31, 33, 35, 39, 51, 52, 56, 58, 59, 66 and 68) in a pooled result [16]. The detection of E6/E7 viral mRNA, which were involved in malignant transformation, provided a marker of active HPV infection associated with high-grade cervical disease and invasive cancer. Cervical specimens in Thin Prep™ Pap test vials containing PreservCyt™ Solution were used for testing with the Aptima HPV 16 18/45 [16].

### Using the HPV RNA Hologic assay

This method was used to test 34.7% (2032/5856) samples which were mainly from the general hospitals. The Aptima HPV 16 18/45 genotype assay involved three main steps, which took place in a single tube: target capture; target amplification by Transcription-Mediated Amplification (TMA) and detection of the amplification products (amplicon) by the Hybridization Protection Assay (HPA) [17]. The assay incorporated an Internal Control (IC) to monitor nucleic acid capture, amplification, and detection, as well as operator or instrument error [17]. Specimens were collected in or transferred to a tube containing specimen transport media (STM) that lysed the cells, released the mRNA, and protected it from degradation during storage [18]. When the Aptima HPV 16 18/45 genotype assay was performed, the target mRNA was isolated from the specimen by use of capture oligomers that were linked to magnetic microparticles [18]. During the detection step, light emitted from the labeled RNA: DNA hybrids was measured as photon signals called Relative Light Units (RLU) in a luminometer [18]. Final assay results were interpreted based on the analyte signal-to-cutoff (S/CO) ratio. IC was added to each reaction via the Target Capture Reagent [18]. The IC monitored the target capture, amplification, and detection steps of the assay. The Dual Kinetic Assay (DKA) was the method used to differentiate the HPV signals and the IC signal [19]. IC and HPV 16 amplicon were detected by probes with rapid light-emission kinetics (flasher) [19]. The IC signal in each reaction was discriminated from the HPV 16 signal by the magnitude of the light emission. Amplicons specific to HPV 18 and 45 were detected using probes with relatively slower kinetics of light emission (glower) [20, 21].

### Data analysis

Quantitative data analysis was conducted using Stata version 15. Frequencies and percentages were calculated for descriptive statistics including HPV prevalence overall and for specific genotypes (HPV 16, 18/45, and other types). To identify factors associated with HPV infection, modified Poisson regression was used to estimate prevalence ratios (PRs) rather than odds ratios, since the outcome prevalence exceeded 10%.

Initially, unadjusted PRs were generated to select candidate variables for inclusion in the multivariate model (*p* < 0.20). Multicollinearity analysis was performed but no variables required removal (*p* < 0.40). The remaining variables were entered into a multivariate modified Poisson regression model to determine adjusted PRs. A forward and backward elimination process was utilized to select the final variables significant at *p* < 0.05.

The Poisson model allowed estimation of PRs instead of odds ratios to avoid overestimation of the associations, given the high HPV prevalence. The stepped modeling strategy identified independent predictors of HPV infection after controlling for confounders.

## Results

### Socio demographic characteristics of participants

Of the 5856 participants, 4439 (75.8%) were between 36 and 49 years, more than half had primary 3188 (54.4%) as their highest education level and over seven in 10 were married 4281 (73.1%). Majority of the participants 4520 (77.2%) had multiple children (multiparous), 3472 (59.3%) had one partner, and 2757 (47.1%) reported early sex (Table 1).

**Table 1:** Socio-demographic characteristics of participants

Variables	Frequency (n)	Percentage (%)
**Age**		
25–35	1417	24.2
36–49	4439	75.8
**Parity/Birth**		
Primiparous	1336	22.8
Multiparous	4520	77.2
**Education level**		
Illiterate	463	7.9
Primary	3201	54.7
Secondary	582	9.9
Tertiary	1610	27.5
**Partners**		
One partner	3472	59.3
More partners	2384	40.7
**Smoking**		
No	5542	94.6
Yes	314	5.4
**Early Sex**		
No	3099	52.9
Yes	2757	47.1
**Marital status**		
not married	1575	26.9
married	4281	73.1
**Occupation**		
Business	1453	24.8
Farmer	1853	31.6
Housewife	1156	19.7
Salarised	1394	23.8
**Machines**		
Genexpert	3824	65.3
Hologic PCR	2032	34.7
**Results**		
Negative	3850	65.7
Positive	2006	34.3
**Genotype**		
None	3850	65.7
HPV − 16	317	5.4
HPV 18/45	308	5.3
Other HPV	1381	23.6
**Districts**		
Fort portal	80	1.4
Gulu	50	0.8
Hoima	809	13.8
Jinja	749	12.8
Kabale	593	10.1
Kyenjojo	381	6.5
Lacor	541	9.2
Lira	902	15.4
Masaka	423	7.2
Mbale	344	5.9
Mbarara	145	2.5
Moroto	147	2.5
Mubende	237	4.1
Naguru	152	2.6
Soroti	303	5.2

### Prevalence of genital high-risk HPV infection among HIV positive women

**Fig. 1:**
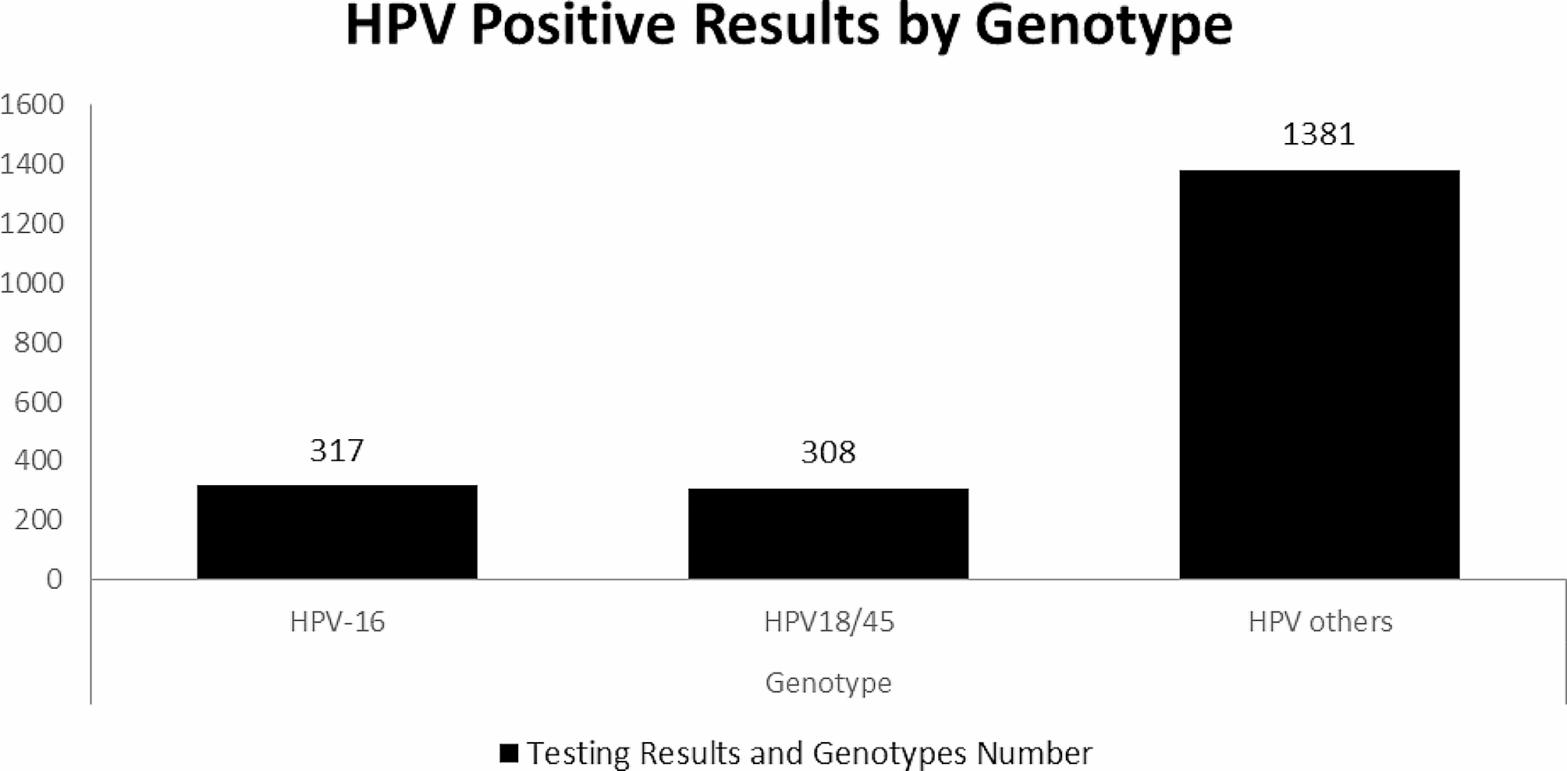
Prevalence of genital high risk HPV infections among study participants

**Fig. 2:**
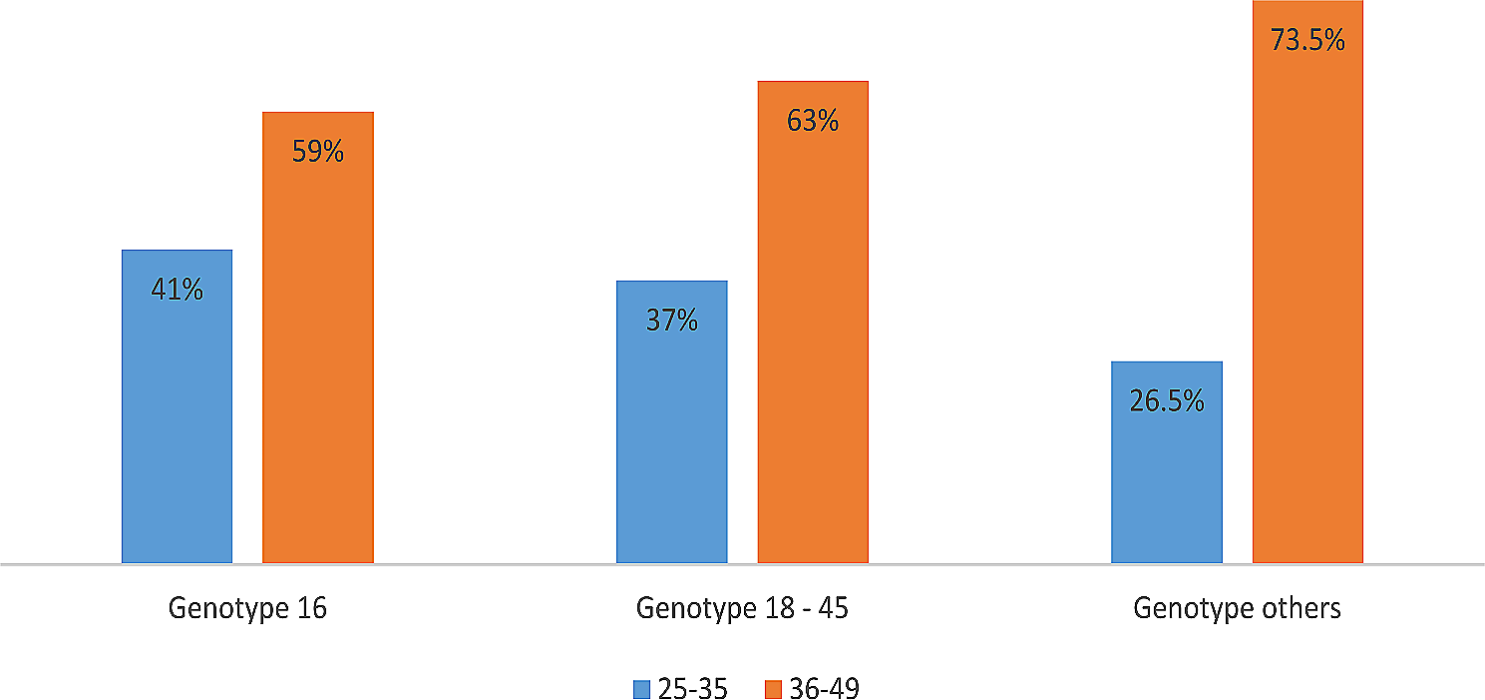
Relative proportion of genital HPV infections (genotypes) among study participants acccording to their age groups

A total of 2006 out of 5856 (34.3%) participants had hrHPV infections as follows: HPV 16 317 (15.8%), HPV 18/45 308 (15.4%) and other hrHPV 1381 (68.8%) (Fig. 1). Genital HPV infections by age distribution showed that all infections were more prevalent among older adults (36–49 years). That is HPV 16 59%, HPV 18/45 63% and other hrHPV 75% (Fig. 2).

### Factors associated with high-risk HPV, HPV 16, HPV 18–45, and other genotypes

At bivariate analysis, across all genital hrHPV infections as well as HPV 16, 18/45 and other genotypes, age, parity, education level, number of partners, marital status, and engaging in early sex were significantly associated with HPV infection except for smoking status that was not associated with HPV 18/45. On the other hand, occupation was only associated with HPV 16 (Table 2).

**Table 2:** Factors associated with all high-risk HPV, HPV 16, HPV 18/45, and other genotypes at bivariate analysis

	All high-risk HPV		HPV 16		HPV 18–45		Others genotypes	
Variables	Yes (%)	UPR (95% CI)	Yes (%)	UPR (95% CI)	Yes (%)	UPR (95% CI)	Yes	UPR (95% CI
**Age**								
25–35	610 (30.4)	1	130 (41)	1	114 (37)	1	366 (26.5)	1
36–49	1396 (69.6)	0.73 (0.68–0.79)***	187 (59)	0.46 (0.37–0.57)***	194 (63)	0.54 (0.43-67)***	1015 (73.5)	0.88 (0.79–0.98)*
**Parity/Birth**								
Primiparous	936 (46.7)	1	255 (80.4)	1	220 (71.4)	1	461 (33.4)	1
Multiparous	1070 (53.3)	0.34 (0.32–0.36)***	62 (19.6)	0.72 (0.05–0.09)***	88 (28.6)	0.12 (0.09–0.15)***	920 (66.6)	0.59 (0.53–0.65)***
**Education level**								
Illiterate	139 (6.9)	1	47 (14.8)	1	13 (4.2)	1	79 (5.7)	1
Primary	1147 (57.2)	1.19 (1.03–1.38)**	172 (54.3)	0.53 (0.39–0.72)***	174 (56.5)	1.93 (1.11–3.37)*	801 (58)	1.46 (1.18–1.81)***
Secondary	174 (8.7)	0.99 (0.83–1.20)	61 (19.2)	1.03(0.72–1.48)	15 (4.9)	0.91 (0.44–1.90)	98 (7.1)	0.98 (0.75–1.29)
Tertiary	546 (27.2)	1.13 (0.97–1.32)*	37 (11.7)	0.23 (0.15–0.34)***	106 (34.4)	2.34 (1.33–4.13)**	403 (29.2)	1.46 (1.17–1.82)***
**Partners**								
One partner	815 (40.6)	1	83 (26.2)	1	85 (27.6)	1	647 (46.8)	1
More partners	1191(59.4)	2.13 (1.98–2.29)*	234 (73.8)	4.11 (3.21–5.24)***	223 (72.4)	3.82 (2.99–4.87)***	734 (53.2)	1.65 (1.51–1.81)***
**Smoking**								
No	1860 (92.7)	1	201 (63.4)	1	297 (96.4)	1	1362 (98.6)	1
Yes	146 (7.3)	1.39 (1.22–1.57)***	116 (36.6)	10.19 (8.35–12.42)***	11 (3.6)	0.65 (0.36–1.18)	19 (1.4)	0.24 (0.15–0.38)***
**Early Sex**								
No	50 (2.5)	1	9 (2.8)	1	14 (4.5)	1	27 (1.9)	1
Yes	1956 (97.5)	43.9 (33.37–57.95)***	308 (97.2)	38.46 (19.86–74.49)***	294 (95.5)	23.6 (13.84–40.25)***	1354 (98.1)	56.36 (38.64–82.22)***
**Marital status**								
not married	789 (39.3)	1	133 (41.9)	1	125 (40.6)	1	531 (38.5)	1
married	1217 (60.7)	0.57 (0.53–0.61)***	184 (58.1)	0.51 (0.41–0.63)***	183 (59.4)	0.53 (0.43–0.67)***	850 (61.5)	0.59 (0.54–0.64)***
**Occupation**								
Business	484 (24.1)	1	58 (18.3)	1	84 (27.3)	1	342 (24.7)	1
Farmer	650 (32.4)	1.05 (0.96–1.16)	119 (37.5)	1.61(1.18–2.18)**	89 (28.9)	0.83 (0.62–1.11)	442 (32)	1.01 (0.89–1.14)
Housewife	394 (19.6)	1.02 (0.92–1.14)	72 (22.7)	1.56(1.11–2.18)*	52 (16.9)	0.77 (0.55–1.09)	270 (19.5)	0.99 (0.86–1.14)
Salarised	478 (23.8)	1.02 (0.93–1.14)	68 (24.5)	1.22(0.86–1.72)	83 (26.9)	1.03 (0.76–1.38)	327 (23.7)	0.99 (0.87–1.13)

* *P* < 0.05, ** *P* < 0.01, ***P < 0.001

At multivariable analysis, compared to women aged 25–35 years, those aged 36–49 had a lower prevalence of all hrHPV [APR = 0.95 (95% Confidence Interval (CI): 0.90–0.99)] and HPV 18–45 [APR = 0.69 (95% CI: 0.56–0.86)]. Compared to uniparous women, being multiparous was associated with lower all hrHPV [APR = 0.85 (95% CI: 0.81–0.89], HPV 16 [APR = 0.19 (95% CI: 0.14–0.25] and HPV 18–45 [APR = 0.29 (95% CI: 0.22–0.39)] and a higher prevalence of the other HPV genotypes [APR = 1.41 (95% CI: 1.29–1.53)]. Regarding education level and compared to those that did not have formal education, having primary and tertiary education was associated with a higher prevalence of all high-risk HPV types (primary [APR = 1.14 (95% CI: 1.04–1.24)]; tertiary [APR = 1.12 (95% CI: 1.01–1.23)], HPV 18–45 (primary [APR = 1.76 (95% CI: 1.04–2.98)]; tertiary [APR = 2.14 (95% CI: 1.25–3.67)] and other genotypes (primary APR = 1.40 (95% CI: 1.19–1.66); tertiary APR = 1.45 (95% CI: 1.22–1.73)). For HPV 16, primary [APR = 0.48 (95% CI: 0.36–0.62), secondary [APR = 0.73 (95% CI: 0.54–0.98)], and tertiary [APR = 0.23 (95% CI: 0.15–0.34)] education were associated with a lower HPV prevalence. A higher number of sexual partners was associated with a higher prevalence of high-risk HPV [APR = 1.9 (95% CI: 1.80–2.04)], HPV 16 [APR = 2.22 (95% CI: 1.70–2.89)], 18–45 [APR = 2.43 (95% CI: 1.85–3.19)] and other high-risk HPV [APR = 1.81 (95% CI: 1.66–1.96)]. Smoking was associated with a higher prevalence of HPV 16 [APR = 4.09 (95% CI: 3.37–4.96)] and a lower prevalence of HPV 18–45 [APR = 0.33 (95% CI: 0.18–0.62)] and other high-risk HPV genotypes [APR = 0.21 (95% CI: 0.13–0.32)]. Early sex was strongly associated with a higher prevalence of hr-HPV [43.9 (95% CI: 32.94–58.65) as well as HPV 16 [APR = 19.08 (95% CI: 8.91–40.86)], 18–45 [APR = 13.22 (95% CI: 7.22–24.21)] and other genotypes [APR = 68.36 (95% CI: 46.33-100.85)]. Women who were married had a lower prevalence of all high-risk HPV [APR = 0.91 (95% CI: 0.86–0.95)] and HPV 16 [APR = 0.75 (95% CI: 0.63–0.91]. Compared to those who engaged in business, housewives had a higher prevalence of HPV 16 [APR = 1.47 (95% CI: 1.09–1.97)] (Table 3).

**Table 3:** Factors associated with high-risk HPV, HPV 16, HPV 18–45, and other genotypes at multivariable analysis

	All high-risk HPV		HPV 16		HPV 18–45		Other HPV	
Variables	Yes (%)	APR (95% CI)	Yes (%)	APR (95% CI)	Yes (%)	APR (95% CI)	Yes (%)	APR (95% CI)
**Age**								
25–35	610 (30.4)	1	130 (41)		114 (37)	1	366 (26.5)	
36–49	1396 (69.6)	0.95 (0.90–0.99)*	187 (59)		194 (63)	0.69 (0.56–0.86)***	1015 (73.5)	
**Parity/Birth**								
Primiparous	936 (46.7)	1	255 (80.4)	1	220 (71.4)	1	461 (33.4)	1
Multiparous	1070 (53.3)	0.85 (0.81–0.89)***	62 (19.6)	0.19 (0.14–0.25)***	88 (28.6)	0.29 (0.22–0.39)***	920 (66.6)	1.41 (1.29–1.53)***
**Education level**								
Illiterate	139 (6.9)	1	47 (14.8)	1	13 (4.2)	1	79 (5.7)	1
Primary	1147 (57.2)	1.14 (1.04–1.24)**	172 (54.3)	0.48 (0.36–0.62)***	174 (56.5)	1.76 (1.04–2.98)*	801 (58)	1.4 (1.19–1.66)***
Secondary	174 (8.7)	1.04 (0.89–1.12)	61 (19.2)	0.73 (0.54–0.98)*	15 (4.9)	1.04 (0.51–2.13)	98 (7.1)	104 (0.84–1.28)
Tertiary	546 (27.2)	1.12 (1.01–1.23)**	37 (11.7)	0.23 (0.15–0.34)***	106 (34.4)	2.14 (1.25–3.67)**	403 (29.2)	1.45 (1.22–1.73)***
**Partners**								
One partner	815 (40.6)	1	83 (26.2)	1	85 (27.6)	1	647 (46.8)	1
More partners	1191 (59.4)	1.9 (1.80–2.04)***	234 (73.8)	2.22 (1.70–2.89)***	223 (72.4)	2.43 (1.85–3.19)***	734 (53.2)	1.81 (1.66–1.96)***
**Smoking**								
No	1860 (92.7)		201 (63.4)	1	297 (96.4)	1	1362 (98.6)	1
Yes	146 (7.3)		116 (36.6)	4.09 (3.37–4.96)***	11 (3.6)	0.33 (0.18–0.62)***	19 (1.4)	0.21 (0.13–0.32)***
**Early Sex**								
No	50 (2.5)	1	9 (2.8)	1	14 (4.5)	1	27 (1.9)	1
Yes	1956 (97.5)	43.9 (32.94–58.65)***	308 (97.2)	19.08 (8.91–40.86)***	294 (95.5)	13.22 (7.22–24.21)***	1354 (98.1)	68.36 (46.33-100.85)***
**Marital status**								
not married	789 (39.3)	1	133 (41.9)	1	125 (40.6)		531 (38.5)	
married	1217 (60.7)	0.91 (0.86–0.95)***	184 (58.1)	0.75 (0.63–0.91)**	183 (59.4)		850 (61.5)	
**Occupation**								
Business	484 (24.1)		58 (18.3)	1	84 (27.3)		342 (24.7)	
Farmer	650 (32.4)		119 (37.5)	1.24 (0.94–1.62)	89 (28.9)		442 (32)	
Housewife	394 (19.6)		72 (22.7)	1.47 (1.09–1.97)*	52 (16.9)		270 (19.5)	
Solarized	478 (23.8)		68 (24.5)	0.89 (0.67–1.19)	83 (26.9)		327 (23.7)	

* *P* < 0.05, ** *P* < 0.01, ***P < 0.001

## Discussion

This study determined the prevalence of genital high-risk HPV infections and associated factors among women living with HIV in selected health facilities in Uganda. We found that about 34.3% of HIV positive women aged 25–49 years attending ART at public health facilities in Uganda had high risk HPV infections. The prevalence of HPV 16 genotype was 317 (15.8%), for HPV 18/45; 308 (15.4%) and 1381 (68.8%) for other hrHPV. The independent factors associated with all hrHPV, and the individual genotypes (16, 18/45 and other hrHPV) were parity, education level, having more than one partner, and engaging in early sex. Smoking was associated with HPV 16, HPV 18/45 and other hrHPV. Age was associated with all hrHPV and HPV 18/45, marital status with all hrHPV and HPV 16, and occupation with HPV 16.

HPV infection is a well-established cause of cervical cancer. Routine HPV screening provides an opportunity to identify women with HPV and facilitate follow-up care as needed. Our study found a relatively low genital hrHPV infection prevalence among HIV infected women, compared to a study conducted in Nigeria showed lower prevalence where only 29% women living with HIV had genital HPV infections, and only 10.6% had genital HPV infections among women that are HIV negative [22]. HIV impacts immune function in ways that can increase susceptibility to persistent HPV infection. Specifically, HIV leads to reduced CD4 + and CD8+, T-cell numbers and function, along with higher regulatory T-cell levels. This dampened, anti-inflammatory state makes it harder to clear HPV infection. Given the high HPV prevalence and immune dysfunction, women with HIV are at substantially increased risk for not only HPV acquisition but also progression to invasive cervical cancer compared to HIV-negative women. Active and persistent HPV infections are more likely during uncontrolled HIV disease [23]. Other research has also found that women living with HIV have a higher incidence and faster progression of cervical precancerous lesions compared to HIV-negative women [24]. Persistent infection with high-risk HPV types, especially HPV16 and 18, is a major risk factor for developing cervical cancer. The increased vulnerability to persistent infection in HIV-positive women further escalates their risk of cervical cancer.

Hausen’s research demonstrated that persistent infection with high-risk types of human papillomavirus (hrHPV) can lead to the development of cervical lesions and abnormalities. His work established hrHPV infection as a necessary factor in the pathogenesis of cervical cancer [25, 26]. Cervical cancer screening should be incorporated into HIV care programs in order to increase early detection and save lives. Using self-collected vaginal samples for HPV testing is a feasible approach to improve screening access and coverage in resource-limited settings. This strategy holds promise for enhancing cervical cancer prevention efforts among women living with HIV in low-income regions. Integrating cervical cancer screening into routine HIV care could help address the elevated burden of cervical cancer morbidity and mortality in this high-risk population [27, 28]..

The modifiable factors that were associated with a high prevalence of all hrHPV and the individual genotypes were high parity, multiple sexual partners, and early sexual debut. These factors have been well documented in literature as associated with HPV infection [29–31] including among HIV positive women [29, 32, 33]. Education about the importance of these risk factors should be conducted among all women to contribute to cervical cancer prevention and control efforts. Besides HPV 16, those with primary and tertiary education level had a higher prevalence of HPV. The association between HPV infection and education level has been established in previous studies [24]. It is possible that those with higher education level are at low risk of HPV infections due to access to information. The other important factor was smoking which was associated with HPV 16, HPV 18/45 and other hrHPV. Smoking is also a well-established risk factor for HPV infection [34–36]. As a modifiable factor, health education and behaviour change programmes should support HPV positive women to cease smoking. Women aged 36–49 years had a lower prevalence of HPV compared to those 25–35 years. Older women are more likely to be married and not have several sexual partners which are key HPV risk factors [31, 32]. Moreover, in this study, being married was associated with a lower HPV prevalence across all hrHPV and the individual genotypes (HPV 16, HPV 18/45 and other hrHPV). The association between occupation and HPV 16 requires further inquiry.

While cervical cancer screening programs exist in most developing countries, they often lack systematic, organized population-based screening. There is a need for both opportunistic and coordinated screening integrated into HIV services to promote early detection. Provider-initiated screening programs could help identify precancerous cervical lesions earlier. Self-collection of urine or vaginal samples for HPV testing is a non-invasive approach that could improve patient compliance with screening. Leveraging these types of strategies that are feasible in resource-limited settings provides a major opportunity to strengthen cervical cancer prevention efforts and reduce the heavy burden of cervical cancer among women living with HIV [37, 38].

This study has some limitations. The cross-sectional design prevented us from prospectively examining whether sexual activity leads to HPV infection and subclinical disease in these women. We also could not assess prior HPV exposure. Additionally, we lacked complete details on antiretroviral treatment regimens, duration, current viral load, and CD4 + counts for the HIV-positive participants. The study population may not reflect all women with HIV.

Another limitation was that the study used the HPV RNA Hologic Assay machine that is not recommended by WHO for diagnosis of HPV. However, it was used because it is a CE marked test [39] that was used for facilities that didn’t have GeneXpert machines because it was the most available test that could be used to screen for cancer. It is recommended that samples for screening cancer among HIV women should be done by the health worker, but this was not done in this study. However, the health workers were used to train the participants on how to collect samples themselves so that they are able to do this even after the study.

However, a strength of our study is the use of HPV molecular assays (DNA or RNA), which are more specific than other tests. Also, with a large sample size, this is the first major study in Uganda to evaluate HPV prevalence in this population. Going forward, we propose that studies: prospectively evaluate HPV outcomes in a population of HIV positive women vs. non- HIV positive women. Also, studies looking at determinants of other important risk factors for HPV pathogenesis and progression such as smoking, multiple sexual partners, high parity and sexual debut among HIV positive women are welcome.

## Conclusions

The prevalence of high-risk HPV genotypes among HIV positive women attending ART clinics in Uganda was high. The modifiable factors that were associated with a high prevalence of all hrHPV and the individual genotypes were high parity, multiple sexual partners, and early sexual debut. The other factors that were associated with HPV infection were smoking and a higher education level were associated with a higher prevalence of hrHPV while older age and being married were protective. The integration of cervical cancer screening in ART programmes remains paramount to support the early detection of cervical cancer. Non-invasive self-collected urine and vaginal sampling for cervical cancer screening are an opportunity to increase screening rates.

## Data Availability

Data are available upon request from the corresponding author.

## Abbreviations

ART: Anti Retro Viral Therapy
DNA: Deoxyribo Nucleic Acid
HIV: Human Immunodeficiency Virus
HPV: Human Papilloma Virus
RNA: Ribo Nucleic Acid
hrHPV: High Risk Human Papilloma Virus
IC: Internal Control
